# Resistant hypertension: diagnosis, evaluation, and treatment a clinical consensus statement from the Thai hypertension society

**DOI:** 10.1038/s41440-024-01785-6

**Published:** 2024-07-16

**Authors:** Pairoj Chattranukulchai, Weranuj Roubsanthisuk, Sirisawat Kunanon, Praew Kotruchin, Bancha Satirapoj, Nattawut Wongpraparut, Sarat Sunthornyothin, Apichard Sukonthasarn

**Affiliations:** 1 Division of Cardiovascular Medicine, Department of Medicine, Faculty of Medicine, King Chulalongkorn Memorial Hospital, Chulalongkorn University, Bangkok, Thailand; 2 Division of Hypertension, Department of Medicine, Faculty of Medicine Siriraj Hospital, Mahidol University, Bangkok, Thailand; 3 Department of Emergency Medicine, Faculty of Medicine, Khon Kaen University, Khon Kaen, Thailand; 4 Department of Internal Medicine, Phramongkutklao Hospital and College of Medicine, Bangkok, Thailand; 5 Division of Cardiology, Department of Medicine, Faculty of Medicine, Siriraj Hospital, Mahidol University, Bangkok, Thailand; 6 Division of Endocrinology and Metabolism, Department of Medicine, Faculty of Medicine, King Chulalongkorn Memorial Hospital, Chulalongkorn University, Bangkok, Thailand; 7 Department of Medicine, Cardiovascular Unit, Faculty of Medicine, Chiang Mai University, and Thai Hypertension Society, Bangkok, Thailand

**Keywords:** Blood pressure, Medication, Resistant hypertension, Statement, Thailand

## Abstract

Resistant hypertension (RH) includes hypertensive patients with uncontrolled blood pressure (BP) while receiving ≥3 BP-lowering medications or with controlled BP while receiving ≥4 BP-lowering medications. The exact prevalence of RH is challenging to quantify. However, a reasonable estimate of true RH is around 5% of the hypertensive population. Patients with RH have higher cardiovascular risk as compared with hypertensive patients in general. Standardized office BP measurement, confirmation of medical adherence, search for drug- or substance-induced BP elevation, and ambulatory or home BP monitoring are mandatory to exclude pseudoresistance. Appropriate further investigations, guided by clinical data, should be pursued to exclude possible secondary causes of hypertension. The management of RH includes the intensification of lifestyle interventions and the modification of antihypertensive drug regimens. The essential aspects of lifestyle modification include sodium restriction, body weight control, regular exercise, and healthy sleep. Step-by-step adjustment of the BP-lowering drugs based on the available evidence is proposed. The suitable choice of diuretics according to patients’ renal function is presented. Sacubitril/valsartan can be carefully substituted for the prior renin-angiotensin system blockers, especially in those with heart failure with preserved ejection fraction. If BP remains uncontrolled, device therapy such as renal nerve denervation should be considered. Since device-based treatment is an invasive and costly procedure, it should be used only after careful and appropriate case selection. In real-world practice, the management of RH should be individualized depending on each patient’s characteristics.

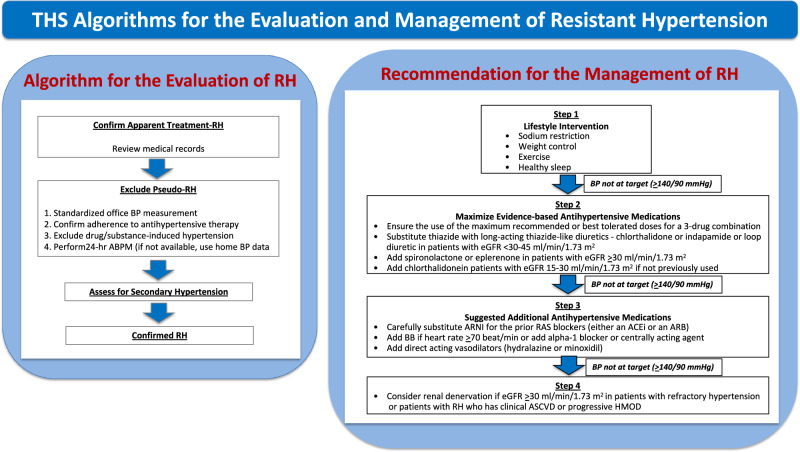

## Introduction

Hypertension is Thailand’s leading risk factor for cardiovascular disease (CVD) and death. Despite the availability of many effective and safe blood pressure (BP)-lowering medications, the rate of achieving BP targets among Thai hypertensive subjects remains low. According to the Thai National Health Examination Survey (NHES), the BP control rate among Thai hypertensive subjects dropped from 61% in 2014 to 48% in 2020 [1, 2]. A nationwide study in Thailand showed that 17% of the Thai hypertensive population was on at least three BP-lowering medications [3].

**Fig. 1:**
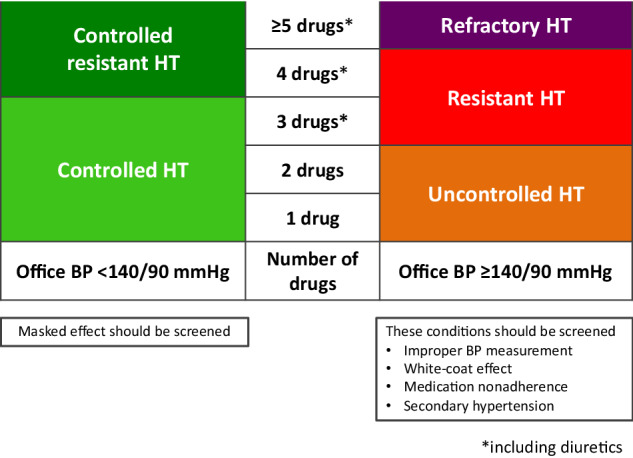
Classification of hypertension according to office blood pressure control and number of antihypertensive drugs

Resistant hypertension (RH) is defined as high BP in a hypertensive patient that remains above goal despite the use of ≥3 antihypertensive agents of different classes, typically including a long-acting calcium channel blocker, a blocker of the renin-angiotensin system (RAS), either an angiotensin-converting enzyme inhibitor (ACEi) or an angiotensin receptor blocker (ARB), and a diuretic, given at maximal or maximally tolerated dose [4, 5]. Classification of hypertension according to office BP control and number of antihypertensive medications is shown in Fig. 1.

RH also includes high BP that is controlled on ≥4 antihypertensive medications, which is controlled RH. The diagnosis of RH requires the exclusion of common causes of pseudoresistance. Therefore, the term apparent treatment-RH is used to indicate patients diagnosed as having RH based on the number of medications used and the uncontrolled office BP (≥140/90 mmHg), but in whom pseudoresistance cannot be excluded (Table 1).

**Table 1:** Definition

Resistant hypertension (RH)	Blood Pressure (BP) that remains elevated above goal in spite of the concurrent use of ≥3 antihypertensive agents of different classes, including a long-acting calcium channel blocker, a blocker of the renin-angiotensin system and a diuretic, administered at maximal or maximally tolerated doses.
Pseudo-RH	A falsely elevated BP in a patient on ≥3 antihypertensive agents caused by improper BP measurement technique, white-coat effect, undertitration of antihypertensive agents, clinical inertia, medication nonadherence, and other factors.
Controlled RH	BP that is controlled on the concurrent use of ≥4 antihypertensive medications at maximal or maximally tolerated doses.
Apparent treatment-RH	BP that remains elevated above goal with the concurrent use of ≥3 antihypertensive agents but medication dose, adherence, or out-of-office BP is not accounted for, and pseudoresistance cannot be excluded.
White-coat RH	Office BP is uncontrolled but out-of-office BP is controlled in a patient on ≥3 proper antihypertensive agents.
Masked RH	Out-of-office BP remains uncontrolled even office BP is controlled in a patient on ≥3 proper antihypertensive agents.
Refractory hypertension	BP that remains uncontrolled despite maximal or near-maximal therapy, with the use of ≥5 antihypertensive agents from different classes, including a long-acting thiazide-like diuretic and spironolactone.

## Prognosis and prevalence of RH

Data from observational studies showed that patients with RH are at higher risk for the development of hypertension-mediated organ damage (HMOD) and poor cardiovascular and renal outcomes compared with hypertensive patients without RH [6–9], suggesting that RH represents a significant public health problem. Uncontrolled BP in RH either confirmed by ambulatory BP monitoring [10] or home BP measurement [11, 12] is associated with higher cardiovascular events, including stroke, coronary artery disease, heart failure, and aortic dissection. Among patients with RH, BP lowering is associated with a reduced risk for most cardiovascular events [7], mainly stroke and coronary heart disease [13].

The prevalence of RH is difficult to quantify because it depends on the clinical setting, the definition of patients’ adherence to treatment, methods, and variations in BP measurement, and the definition of the BP target representing controlled BP. Using a BP target of <140/90 mmHg and excluding patients with normal out-of-office BP values, verifying medication adherence, and excluding secondary hypertension, a reasonable estimate of the true RH should be about 5% of the overall hypertensive population [14].

The prevalence of RH in Thailand has never been reported. In a nationwide survey conducted from 2014 to 2015 involving 65,667 patients with hypertension from 833 hospitals, 11,229 patients (17.1%) were on ≥3 BP-lowering medications [3]. Among this population, 69% achieved the BP target (systolic BP < 140 mmHg and diastolic BP < 90 mmHg), and 31% were not at target. Therefore, the estimated apparent treatment-RH among the Thai hypertensive population should be 5.3% of the treated population. There were reports estimating that the prevalence of pseudo-RH is between 33 and 37% among patients with apparent treatment-RH. Suppose the same is true in the Thai population. In that case, the estimated true RH in the Thai hypertensive population should be around 3.4%, lower than the estimation from a meta-analysis of observational studies in other populations [14]. Data from Malaysia, 24% of adults referred from primary care clinics due to uncontrolled hypertension met the criteria of true RH, confirmed by ambulatory BP monitoring [15]. Furthermore, 26.6% of those RH subjects were defined as refractory hypertension, elevated BP despite treatment with at least 5 antihypertensive medications [15]. This indicates that the prevalence of RH amongst Asian adults with uncontrolled hypertension might be high.

Contributing clinical factors of RH include obesity, excessive alcohol consumption, high sodium intake, and advanced atherosclerotic disease with HMOD. Patients with RH also have a higher prevalence of comorbid conditions, including diabetes mellitus, chronic kidney disease (CKD), coronary heart disease, and stroke [8].

It is uncertain whether the increased cardiovascular risk seen in RH is related solely to the persistent elevation of BP per se or whether other specific pathophysiologic factors, such as increased renin-angiotensin and sympathetic nervous system activity, hyperaldosteronism, and increased arterial stiffness, may also involve. Data showing that BP lowering may confer less improvement in the cardiovascular risk profile of patients with RH than in hypertensive patients without RH suggest that the presence of RH is also an important predictor of CVD risk more than the level of BP alone [13].

## Diagnostic work-up

The evaluation of patients with apparent treatment-RH should be directed toward excluding pseudo-RH, which requires (1) standardized office BP measurement (Table 2) (2) confirmation of adherence to antihypertensive therapy (3) exclusion of drug- or substance-induced RH (Table 3) (4) data from 24-h ambulatory BP monitoring (if not available, use home BP monitoring) to exclude the white-coat effect.

**Table 2:** Standardized office blood pressure measurement

1. Patients should sit on a chair with back support, rest both feet on the floor, and relax for at least 5 min in a quiet room with comfortable temperature and an empty bladder. They should not have consumed caffeine, smoked, or exercised in the last 30 min.
2. Use a validated upper-arm blood pressure measuring device that has been properly calibrated and a properly sized arm cuff.
3. Take measurements on bare skin in both upper arms, with the patient’s upper arm supported. Use the arm with the higher reading for subsequent measurements, and repeat the measurements 3 times, 1-min apart. There should be no conversation before, during, or between measurements.
4. Use the average of at least 2 readings from measurement to estimate blood pressure.

**Table 3:** Etiologies of secondary hypertension based on organ systems

Endocrine causes	• Primary aldosteronism • Pheochromocytoma/paraganglioma • Cushing syndrome • Hyperthyroidism and hypothyroidism • Hypercalcemia and primary hyperparathyroidism • Congenital adrenal hyperplasia • Acromegaly
Metabolic disease	• Obstructive sleep apnea
Renal causes	• Renal parenchymal disease e.g., glomerulonephritis, polycystic kidney disease, and chronic kidney disease • Renal artery stenosis
Cardiovascular causes	• Coarctation of aorta
Drug or substance causes	• Nonsteroidal anti-inflammatory drugs (NSAIDs) • Selective cyclooxygenase-2 (COX-2) inhibitors • Oral contraceptives • Glucocorticoids and mineralocorticoids • Sympathomimetic amines: amphetamine, ephedrine, pseudoephedrine, phenylpropanolamine • Antidepressants • Erythropoietin-stimulating agents • Alcohol • Cocaine • Cyclosporine, tacrolimus • Vascular endothelial growth factor (VEGF) inhibitors • Herbal products

After confirming RH, assessment for secondary hypertension, which can partly be guided by medical history and physical examination, is required (Table 3).

Physical examination of a patient with RH requires ascertainment of HMOD and possible clues for secondary causes. The BP should be measured carefully on both arms, as described in Table 2. Fundoscopic evaluation and examination of carotid and peripheral pulses are necessary. Abdominal bruits, if detected, can suggest the possibility of obstructive renal artery disease. General appearances and stigmata that can indicate hypercortisolism, hyperthyroidism, hypothyroidism, pheochromocytoma, and acromegaly should be carefully examined. Among the secondary causes of RH, primary aldosteronism is one of the most common causes and may not have suggestive clues from initial history taking, physical examination, and laboratory tests. Therefore, primary aldosteronism is often underdiagnosed, and every patient with RH should be screened for primary aldosteronism with the measurement of plasma renin and aldosterone levels [16]. If there is any suspicion of any causes of secondary hypertension, specific investigations should be pursued. However, if further investigations cannot be done, referral to a tertiary care center is recommended. Still, this should ensure that the intensification of the BP-lowering treatment regimen is not delayed.

**Fig. 2:**
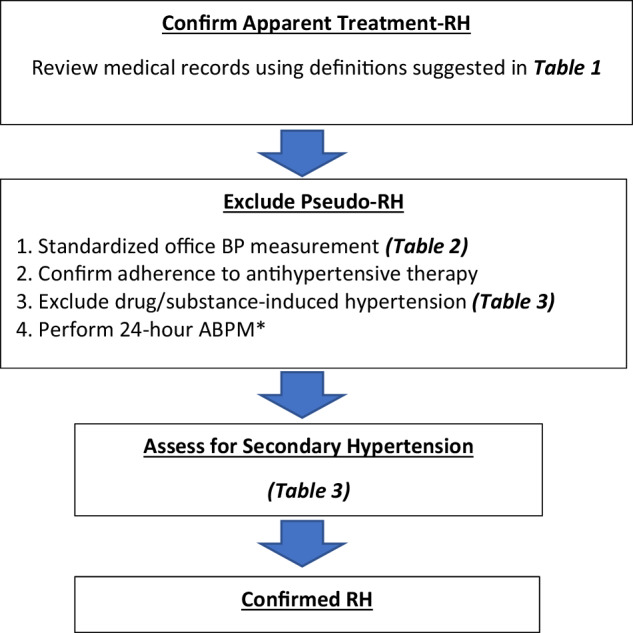
Algorithm suggested for evaluation of resistant hypertension. *If not available, home blood pressure monitoring can be used. RH resistant hypertension, BP blood pressure, ABPM ambulatory blood pressure monitoring

The algorithm suggested for the evaluation of RH is shown in Fig. 2.

## Management of RH

Effective treatment of RH requires lifestyle modification, along with a stepwise approach using antihypertensive medications with different mechanisms of action, and considering device-based treatment when necessary.

**Fig. 3:**
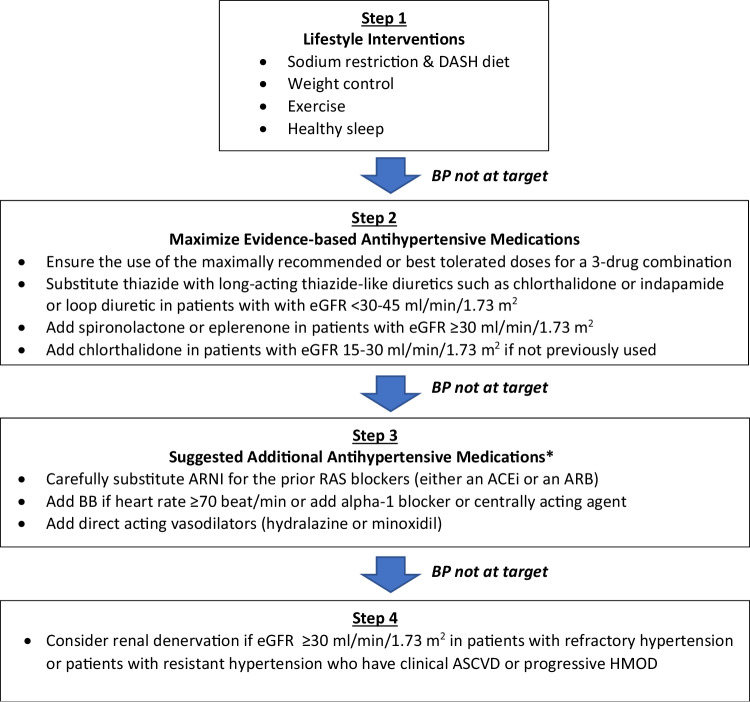
Recommendations for the management of resistant hypertension. *The choice depends on clinical circumstances, drug availability, and physician preferences. eGFR estimated glomerular filtration rate, ARNI angiotensin-receptor-neprilysin-inhibitor, RAS renin-angiotensin system, ACEi angiotensin-converting enzyme inhibitor, ARB angiotensin receptor blocker, BB beta-blocker, ASCVD atherosclerotic cardiovascular disease, HMOD hypertension-mediated organ damage

A suggested algorithm for managing RH is shown in Fig. 3.

## Nonpharmacological and non-antihypertensive pharmacological treatment

Dietary sodium restriction has been well-proven to decrease BP [17]. In addition, subgroups of hypertensive individuals, such as patients with CKD and obesity, are often more sensitive to sodium intake and can benefit more in BP reduction from sodium restriction [18, 19]. Patients with RH usually experience a greater BP reduction from the limitation of sodium intake than other patients with hypertension, suggesting that salt sensitivity may play an essential role in the pathogenesis of RH.

Patients should be recommended to consume less than 2 g of sodium per day (<5 g of sodium chloride), in addition to following the Dietary Approaches to Stop Hypertension (DASH) diet, which is rich in fruits, vegetables, and low-fat dairy products. Combining these two strategies is more effective than either alone [20].

Diet control and exercise have been proven to significantly lower office and ambulatory BP in patients with RH, according to a recent randomized clinical trial, which also demonstrated improvement in selected CVD biomarkers in this population [21]. In another recent randomized trial, a 12-week moderate-intensity aerobic exercise training program can reduce 24-h, daytime ambulatory BP, and office systolic BP in patients with RH [22].

Various nonpharmacological interventions (e.g., meditation, stress-reducing therapies, augmentation of dietary potassium, and using potassium as a sodium substitute) have also been reported to lower BP. However, the extent and quality of the supporting clinical trials in patients with RH are not enough to recommend their applications in this specific group of hypertensive patients.

Weight reduction through diet control is well-established to lower BP among patients with hypertension [23]. However, this needs to be adequately evaluated in patients with RH. On the other hand, although the effects of many new pharmacologically aided weight loss options, such as glucagon-like peptide-1 receptor agonist (GLP-1 RA) and sodium-glucose cotransporter 2 (SGLT2) inhibitors, on BP reduction, are promising, there is not enough data to recommend their use in patients with RH.

Bariatric surgery is an effective strategy to reduce BP and improve metabolic risk factors in morbidly obese patients. This treatment is invasive and there is insufficient data to suggest its role in RH [24].

We recommend weight loss through diet control and regular exercise, which provide overall cardiovascular and metabolic benefits and likely reduce BP in patients with RH [21–23].

Obstructive sleep apnea (OSA) is common in patients with RH, especially in male patients [25]. RH and OSA are hypothesized to share a common mechanism: an excess of aldosterone [26, 27]. This hypothesis is supported by studies demonstrating that spironolactone and eplerenone can reduce the severity of OSA in patients with RH [28–30]. However, the use of continuous positive airway pressure (CPAP) to treat OSA can induce only a modest decrease in BP in patients with RH [31]. Because OSA is common in patients with RH, these patients should be screened for the possibility of OSA, and when detected, they should be treated with CPAP. The benefit of CPAP in these patients is better when the patients are fully adherent to CPAP (4–8 h/night) [31].

## Pharmacological treatment

Pharmacological therapy of RH is based on the use of ≥3 antihypertensive medications. These should include a RAS blocker (an ACEi or an ARB), a long-acting calcium channel blocker, and a diuretic. In patients with CKD, consider transitioning the diuretic to a loop diuretic if the estimated glomerular filtration rate (eGFR) is <30–45 ml/min/1.73 m^2^. Many antihypertensive agents are now available in various dual or triple-pill combinations, allowing for simplified regimens. However, to ensure that the patients receive the maximally recommended or maximally tolerated doses of all three agents, some single or double components of the three medications must be added. Also, if the prescribed diuretic is a thiazide, it should be replaced by a long-acting thiazide-like diuretic, preferably chlorthalidone or indapamide. All these modifications usually result in the prescription requiring at least two or three pills instead of just a single combination pill.

In patients with RH, the fourth medication should include a mineralocorticoid receptor antagonist (MRA), specifically spironolactone, based on the PATHWAY-2 clinical trial [32]. Spironolactone (25–50 mg/day) should be used cautiously in patients with an eGFR <45 ml/min/1.73 m^2^ and a plasma potassium >4.5 mmol/l. We recommend adding spironolactone or eplerenone [33] only in patients with RH and eGFR ≥30 ml/min/1.73 m^2^. If not previously used, chlorthalidone should be added in patients with RH and eGFR 15–30 ml/min/1.73 m^2^ (Fig. 3).

Spironolactone carries a significant risk of hyperkalemia, especially in patients with CKD, particularly if the drug is added to a treatment regimen that usually includes a RAS blocking agent. It is necessary to closely monitor plasma potassium and renal function within 8 weeks after treatment initiation. Not all patients will tolerate spironolactone well due to its antiandrogenic adverse effects, resulting in gynecomastia, breast tenderness, and sexual impotence in men, and its progestogenic effect, resulting in menstrual irregularities in women. An alternative drug is amiloride at a high dosage (10–20 mg per day), which is as effective as spironolactone (25–50 mg per day). However, this drug is not available in the Thai market. If spironolactone is not tolerated or contraindicated, chlorthalidone can be considered, even in patients with eGFR ≥30 ml/min/1.73 m^2^, but previously used diuretic must be discontinued first.

A long-acting thiazide-like diuretic, specifically chlorthalidone, has superior efficacy compared to hydrochlorothiazide. There have been reports demonstrating that substituting chlorthalidone for hydrochlorothiazide in patients with uncontrolled hypertension on multiple-drug combinations provides further BP reduction and improves overall control rates [34]. Additionally, there was a report showed that chlorthalidone 12.5–50 mg/day can improve BP control among patients with advanced CKD and poorly controlled wit despite receiving a mean (±SD) number of 3.4 (±1.4) antihypertensive medications [35].

Sacubitril/valsartan, a first-in-class dual-action molecule angiotensin-receptor-neprilysin-inhibitor (ARNI), has recently been approved for treating high BP in Thailand. Sacubitril/valsartan was shown in a double-blind randomized controlled trial to be superior to olmesartan in lowering office and ambulatory central aortic and brachial BP in elderly patients with systolic hypertension and stiff arteries (pulse pressure >60 mmHg) [36]. A meta-analysis of 11 randomized clinical trials in 6,028 participants demonstrated that sacubitril/valsartan was superior to ARBs for treating hypertension [37].

The post hoc analysis of the PARAGON-HF (the Prospective Comparison of ARNI with ARB Global Outcomes in Heart Failure with Preserved Ejection Fraction) trial [38] showed that 731 patients (15.2%) with heart failure with preserved ejection fraction (HFpEF) had apparent treatment-RH and 135 (2.8%) had apparent MRA-RH [39]. In this analysis, the reduction in systolic BP after 1 and 4 months was greater with ARNI than with valsartan in patients with RH and was even greater in patients with MRA-RH. Synergistic mechanisms of action could explain stronger reduction in systolic BP among MRA-RH patients: spironolactone targets sodium and water retention, and neprilysin inhibition targets natriuretic peptide. Sacubitril/valsartan also significantly reduces arterial stiffness more than ARB, which suggests more beneficial results in patients with RH [40].

Neprilysin inhibition depends on biologically active natriuretic peptides and their binding with particulate guanylate cyclase/cyclic guanylate monophosphate-coupled receptor. This induces vasodilation, decreases vascular stiffness, reduces oxidative stress, and induces diuresis and natriuresis [41]. The combination of valsartan and sacubitril could also reduce the free radicals, lower the levels of tumor necrosis factor-alpha and proinflammatory cytokines that are responsible for renin-angiotensin-aldosterone system and sympathetic nervous system overactivation, fibrosis, vascular and microvascular dysfunction, and left ventricular diastolic dysfunction. From a clinical perspective, ARNI should be helpful in patients with RH, especially those with HFpEF.

If the patient requires switching from an ACEi or an ARB to an ARNI, we suggest discontinuing an ACEi for at least 36 h before starting ARNI to prevent angioedema. However, after discontinuation of an ARB, ARNI can be started immediately.

If the BP is still uncontrolled after spironolactone or chlorthalidone, or if the drugs are not tolerated, other alternative treatments besides ARNI at this step are beta-blockers or combined alpha-beta-blockers (carvedilol), doxazosin extended-release (4–8 mg per day) [42], or a centrally acting agent such as clonidine or methyldopa. The choice depends on clinical circumstances, drug availability, and physician preferences.

A direct vasodilator, such as hydralazine or minoxidil, should be added if BP is still uncontrolled. They can cause fluid retention and reflex tachycardia, so they should be used only when clinically indicated. Minoxidil can also cause hypertrichosis.

## Device-based treatment

Few patients with RH still have uncontrolled hypertension despite using full medications and having good adherence. Some of these patients may develop adverse reactions to various BP-lowering drugs, and the medications cannot be up-titrated to the required maximum doses. In these scenarios, consideration of device-based treatment is necessary. The device-based treatment that has been widely confirmed to be effective in RH is catheter-based renal denervation (RDN) therapy [43–45] with 24-h BP-lowering and long-term durability [46–50]. To date, numerous clinical trials have proven that RDN can lower BP in patients with RH and even with refractory hypertension. However, the BP reduction after RDN can vary among patients. Since RDN is an invasive and costly treatment, we suggest using RDN only after careful and appropriate patient selection, as indicated in a recent Thai Hypertension Society statement [51].

## Conclusions

RH remains a challenging aspect in managing subjects with hypertension. True RH’s estimated prevalence in Thai hypertensive populations should be around 3.4% of the treated population. Considering the increased prevalence of hypertension in Thailand in recent years, the number of patients with RH may be significantly increasing. A stepwise approach starting with carefully standardized office and out-of-office BP measurements, exclusion of drugs and substances that may elevate BP, and workup for secondary causes are mandatory. Intensive lifestyle modification, together with enhanced medication adherence, is also crucial. The application for appropriately selected with a maximally tolerated dosage of antihypertensive medications is suggested, including sacubitril/valsartan as one of the fourth and subsequent lines of therapy. The treatment of RH should be individualized depending on the patient’s characteristics, and the medication used in each personalized combination should be carefully chosen. Since all treatments must be continued for a long time, considering long-term adverse effects and compliance with all regimens is extremely important. These measures are not always successful in true RH, and some patients may require catheter-based RDN.
